# Research Progress on the *Dendrolimus* spp. Pheromone: From Identification to Molecular Recognition

**DOI:** 10.3389/fcell.2022.829826

**Published:** 2022-05-03

**Authors:** Sufang Zhang, Xiangbo Kong, Zhen Zhang

**Affiliations:** Key Laboratory of Forest Protection of National Forestry and Grassland Administration, Ecology and Nature Conservation Institute, Chinese Academy of Forestry, Beijing, China

**Keywords:** forest pests, periodic outbreak, pheromone, PBP, PR

## Abstract

*Dendrolimus* species (Lepidoptera, Lasiocampidae), are the most serious phytophagous pests of coniferous forests worldwide. *Dendrolimus* feed intensively on needles, leading to considerable economic loss and ecological damage. Notably, the outbreak of *Dendrolimus* is a somewhat periodic pattern, and those outbreaks cause rapid and large-scale destruction of pine forests, with those forests observed to look like “Fire without smoke”. Sex pheromones play an important role during insect mating and reproduction, and there has been extensive research into the pheromone of *Dendrolimus*. The pheromone components of several *Dendrolimus* have been identified, and functions of two most important pheromone recognition genes, pheromone-binding proteins (PBPs) and pheromone receptors (PRs), were clarified. The evolution of PBP gene sequences is in good agreement with the trends in structural changes of the sex pheromone components in several *Dendrolimus* species, and it is interesting that PRs of *Dendrolimus* spp. occupy a novel lineage of PRs tuned to Type I pheromones in Lepidoptera. We present the current state of research into the sex pheromone of these important forest pests and highlight the emerging topics, to clarify future urgent work into *Dendrolimus*.

## 1 Introduction

*Dendrolimus* spp. (Lepidoptera, Lasiocampidae) are the most serious phytophagous pests of coniferous forests worldwide. More than 30 species in the genus *Dendrolimus* are identified around the world of which about 27 species are present in China (Hou, 1987). The most seriously damaging *Dendrolimus* in China consist of six species including *Dendrolimus punctatus* (Walker), *D. superans* (Butler), *D. tabulaeformis* Tsai et Liu, *D. spectabilis* Butler, *D. houi* lajonquière, and *D. kikuchii* Matsumura. Some *Dendrolimus* species cause damage multiple times a year, with young larvae of the filial generation in summer voraciously feeding on the pine trees. Their damage causes a considerable reduction in standing volume, annual increment, and resin productivity of the trees. In particular, the damaged forests are susceptible and vulnerable to subsequent diseases and insect pests, and even forest fire, all which lead directly to extensive economic loss and ecological disaster (Chen, 1990). Notably, the outbreaks of *Dendrolimus* are somewhat periodic (Zhang et al., 2002). Catastrophic outbreaks usually occur within a short period, and large-scale destruction of pine forests has been referred to as making those forests look like “fire without smoke” (Han et al., 2004). In addition, people and livestock can suffer from serious health problems because of the toxic/irritating hairs on the larvae and cocoons (Hou, 1987). However, controlling *Dendrolimus* with pesticides has impacted human health, the environment, the natural enemies of *Dendrolimus*, and biodiversity (Kong X. B. et al., 2007).

Concomitant with their threats to pine forests and ecosystems, great efforts have been devoted to developing sustainable control strategies for pine caterpillars. Generally, it is not easy to monitor and control forest pests due to the thickness of the forests and the presence of surrounding mountains, so pheromone detection and control are particularly important. As lepidopteran insects, sex pheromones were emitted by *Dendrolimus* females, and the males are highly sensitive to pheromones. For this reason, research into the pheromones of *Dendrolimus* has attracted considerable attention. This review focuses on the advances made in the identification and recognition of *Dendrolimus* spp. pheromones, as well as the further understanding needed to manage these forest pests more effectively.

## 2 Identification of Sex Pheromones of *Dendrolimus* spp.

### 2.1 Pheromone Structure Identification of *Dendrolimus* spp.

Structure identification of pheromone compounds is the first step when researching insect pheromones. Such work in this field began in the 1930s. After years of research, Butenandt et al. (1959) isolated and identified the chemical structure of the first insect sex pheromone *E*8,*Z*10-16: OH, named “bombykol”, from more than 500,000 female silkworm moths. Since then, research into insect pheromone identification has developed rapidly. The application of gas chromatography (GC), gas chromatography–mass spectrometry (GC-MS), gas chromatography–electroantennogram (GC-EAD), high-pressure liquid chromatography (HPLC), other high-performance microanalyzers, and the improvement of separation and extraction technology of insect pheromones has reduced the number of insects needed for the identification of insect pheromones from hundreds of thousands of individuals to just hundreds or even dozens, expediting research in this area.

So far, the sex pheromones of seven *Dendrolimus* species have been elucidated, six of which consist of isomers of (5*Z*,7*E*)-dodecadien-1-ol (*Z*5,*E*7-12:OH), and/or the corresponding acetates, propionates, or aldehyde derivatives (Ando et al., 1982; Priesner et al., 1984; Kovalev et al., 1993; Klun et al., 2000; Kong et al., 2003; Kong X.-B. et al., 2007; Kong et al., 2011; Kong et al., 2012). The seventh species, *D. houi*, uses (5*E*,7*Z*)-dodecadien-1-ol (*E*5,*Z*7-12:OH) and the corresponding acetate and aldehyde as sex pheromone components (Kong X. B. et al., 2007). All the identified sex pheromone components in *Dendrolimus* spp. share a common chemical theme of being C_12_ 5,7-dienes with alcohol, acetate, propionate, or aldehyde functional groups. The brief history of sex pheromone identification of *Dendrolimus* spp. is summarized as follows.

Pheromone identification of *Dendrolimus* began in the 1970s. The sex pheromone of *D. punctatus* was the first one identified from the genus *Dendrolimus*. The insect hormone research group including three groups from the Institute of Zoology, Chinese Academy of Sciences; Jilin Institute of Applied Chemistry, Chinese Academy of Sciences; and the insect group of the Jiangxi forest pest control station, jointly identified the components of the pheromone of *D. punctatus*. The active components of the gonadal extract of *D. punctatus* were preliminarily obtained by extraction and separation; the compounds of the sex pheromone of *D. punctatus* were identified by GC-MS and characteristics MS peak analysis; the position of unsaturation of the aforementioned compounds were confirmed to be located at positions 5 and 7 by micro ozonolysis. Then, EAG (electroantennogram) reactions of single unsaturated dodecenol and its acetate to *D. punctatus* were analyzed, showing that the 5-position double bond could be a cis type, but the cis\trans structure of the 7-position double bond could not be determined. Various isomers (cis\trans, trans\cis, cis\cis, and trans\trans) of 5, 7-dodecadienol and its acetate were then synthesized, and the EAG activity of the antennae of male *D. punctatus* was tested. The results indicated that the cis-5-trans-7-isomers are the components of the pheromone of *D. punctatus*. Up to this point, the structure of the pheromone of *D. punctatus* had been analyzed step by step (Laboratory of Insect Pheromone, Institute of Zoology, Academia Sinica, Pine Caterpillar Moth Pheromone Section, Jilin Institute, of Applied Chemistry, Academia Sinica and Entomology Section, Forest Pest Control Experimental Station, Jiangxi, 1979). The active components of the pheromone gland extract of *D. punctatus* were identified as *Z*5,*E*7-12:OH, (5*Z*,7*E*)-5,7-dodecadien-1-yl acetate (*Z*5,*E*7-12:OAc), and (5*Z*,7*E*)-5,7-dodecadien-1-yl propionate (*Z*5,*E*7-12:OPr) (Laboratory of Insect Pheromone, Institute of Zoology, Academia Sinica, Pine Caterpillar Moth Pheromone Section, Jilin Institute, of Applied Chemistry, Academia Sinica and Entomology Section, Forest Pest Control Experimental Station, Jiangxi, 1979). This important progress demonstrated that researchers of insect pheromones in China were producing work of an international level at that time. Later, (5*Z*)-5-dodecen-1-yl acetate (*Z*5-12:OAc) and (5*Z*)-5-dodecen-1-ol (*Z*5-12:OH) were also found in the sex pheromone gland of *D. punctatus*, two compounds that, when added to the three-component sex pheromone blend, can increase the ease of trapping the *D. punctatus* moth (Zhao et al., 1993).

**FIGURE 1:**
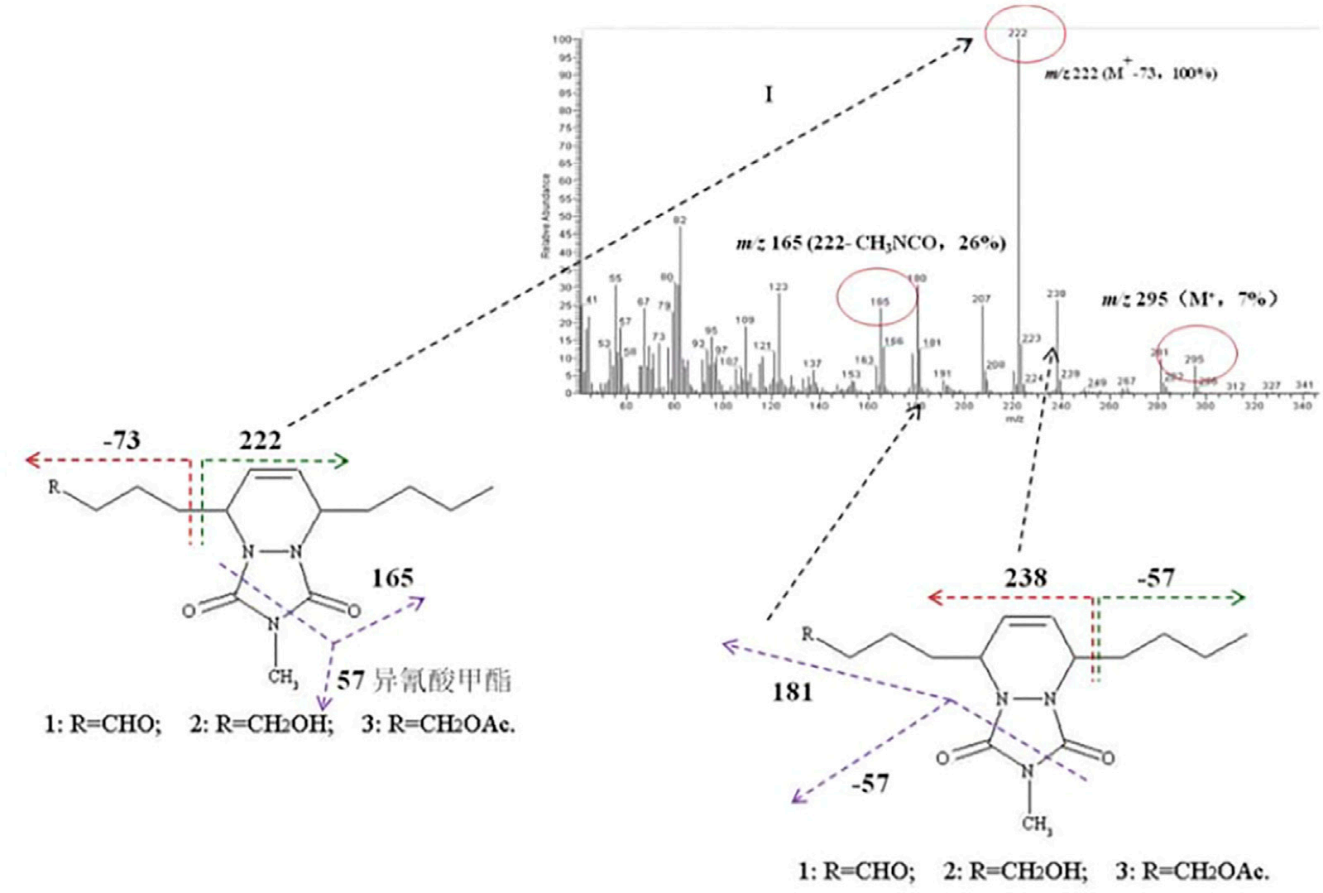
Mass spectrometry output of the derivatives of the sex components of (5*E*,7*Z*)-dodecadien-1-ol and 4-methyl-1,2,4-triazoline-3,5-dione, together with the cleavage law of characteristic fragmentations.

Subsequently, the sex pheromones of several other *Dendrolimus* species were identified. The sex pheromone components of *D. spectabilis* were identified as *Z*5,*E*7-12:OH by Japanese scholars (Ando et al., 1982), and *Z*5,*E*7-12:OAc and *Z*5,*E*7-12:OPr as minor pheromone components by Chinese scholars who have demonstrated that this strong moth lure is composed of these three components at a ratio of 100:3:25 (Kong et al., 2003). The sex pheromone components of *D. pini* were identified as *Z*5,*E*7-12:Ald (Priesner et al., 1984) but completed by Kovalev et al. (1993) who added *Z*5,*E*7-12:OH as a minor pheromone component (Kovalev et al., 1993). The sex pheromone components of *D. superans* were identified as *Z*5,*E*7-12:Ald and *Z*5,*E*7-12:OH at a ratio of 100:98 (Kong X.-B. et al., 2007), which clarified the active components and their isomers (Klun et al., 2000). In 2007, through pheromone gland extraction, GC-MS structure analysis (Figure 1), EAG activity tests, and field trapping verification, the pheromone components of *D. houi* were identified as *E*5,*Z*7-12:OH, *E*5,*Z*7-12:OAc, and *E*5,*Z*7-12:Ald, at a ratio of 100:39.7:5.6, and these chemicals are the first (*E*.*Z*)-isomer sex pheromones found in species of the genus *Dendrolimus*, the other species all being the (*Z*,*E*)-isomer (Kong X. B. et al., 2007). In 2011, the female sex pheromone of *D. kikuchii* was found to be *Z*5,*E*7-12:OAc, *Z*5,*E*7-12:OH, and *Z*5-12:OAc with an optimal attraction ratio of 100:20:25. In 2012, the sex pheromone components of *D. tabulaeformis* were identified as *Z*5,*E*7-12:OAc, *Z*5,*E*7-12:OH, and *Z*5,*E*7-12:OPr, with a ratio of 100:100:4.5 providing the optimal attraction (Kong et al., 2012).

**FIGURE 2:**
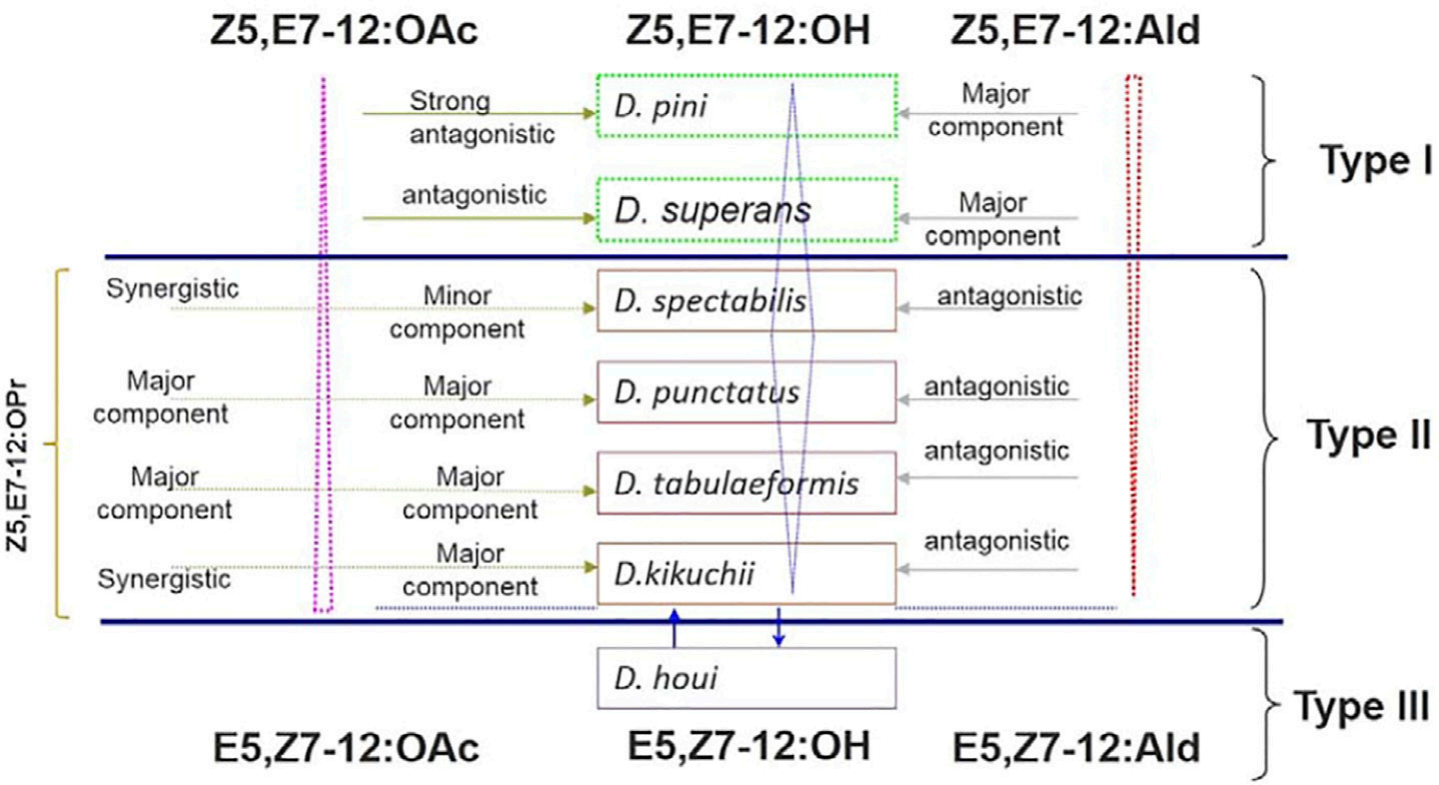
Relationships of sex pheromone components in *Dendrolimus* spp. Dotted pink triangle, blue diamond, and dotted red triangle directed the ratio and function of three important sex pheromone components, *Z*5,*E*7-12:OAc, *Z*5,*E*7-12:OH, and *Z*5,*E*7-12:Ald, respectively.

The relationships between sex pheromone components and *Dendrolimus* species are interesting. The sex pheromone of *D. pini* and *D. superans* belongs to the first sex pheromone type, which uses *Z*5,*E*7-12:Ald as major component without any ester components involved. *D. punctatus*, *D. tabulaeformis*, *D. spectabilis*, and *D. kikuchii* belong to the second sex pheromone type containing *Z*5,*E*7-12:OAc, *Z*5,*E*7-12:OH, and *Z*5,*E*7-12:OPr, but no aldehyde components. It is interesting that *D. punctatus*, *D. tabulaeformis*, and *D. spectabilis* all contain the same pheromone components but with different ratios. Finally, the sex pheromone components of *D. houi* are composed of *E*5,Z7-dodecadienol, and its aldehyde and acetate derivatives, representing the third sex pheromone type in *Dendrolimus* spp. (Figure 2). Based on the content changes of major sex pheromone components of different *Dendrolimus* species, it is evident that they follow certain rules: first, *Z*5,*E*7-12:Ald is the main pheromone component of *D. pini* and accounts for half of the total pheromones of *D. superans*; on the contrary, its potential as a sex pheromone inhibitor in *D. punctatus*, *D. spectabilis*, and *D. kikuchii* gradually strengthened (Figure 2, directed in red dotted triangle). Second, the ratio of *Z*5,*E*7-12:OH, which is a oxidation product of *Z*5,*E*7-12:Ald, in the pheromone mixtures of several *Dendrolimus* were directed in blue diamond. It is the most important sex pheromone component of *D. spectabilis*, *D. punctatus*, and *D. tabulaeformis*, but minor component of *D. pini*, *D. superans*, and *D. kikuchii* (Figure 2). At Last, *Z*5,*E*7-12:OAc, which is a acetate ester of *Z*5,*E*7-12:OH, is major sex pheromone component of *D. punctatus*, *D. tabulaeformis*, and *D. kikuchii*, minor pheromone component of *D. spectabilis*, antagonist of *D. superans*, and strong antagonist *D. pini* (Figure 2, directed in pink dotted triangle).

### 2.2 Synthesis and Application of the Sex Pheromones of *Dendrolimus* spp.

Along with the identification of sex pheromones, synthesis research *in vitro* and *in vivo* was also undertaken. The biosynthesis pathway of the sex pheromone in *D. punctatus* was gradually clarified (Liénard et al., 2010), which is affected by the pheromone biosynthesis activating neuropeptide (PBAN) produced in the brain and subesophageal ganglia complex (Zhao et al., 2002). Meanwhile, the *in vitro* chemical synthesis of the *Dendrolimus* sex pheromone components was developed and applied in field population monitoring (Laboratory of Insect Pheromone, Institute of Zoology, Academia Sinica, Pine Caterpillar Moth Pheromone Section, Jilin Institute, of Applied Chemistry, Academia Sinica and Entomology Section, Forest Pest Control Experimental Station, Jiangxi; Meng and Wang, 1983; Khrimian et al., 2002). Insect pheromone research is a subject closely aligned with practical application. Most of the pheromones or attractants identified and synthesized are from major agricultural or forestry pests, which play an important role in integrated pest management.

When synthetic insect pheromones are used in the field, it is necessary to select an appropriate carrier in order to achieve a stable release of the semiochemicals. The most widely used carriers are made of polyethylene plastic pipe, sleeve rubber, plastic film, microcapsules, etc. The release rates of *Dendrolimus* sex pheromone components on different carriers were evaluated (Li et al., 2015). The sulfur-free composite rubber carrier has good anti-isomerization ability and slow-release performance (Gao et al., 2001). The primary application of sex pheromones in pest control is population monitoring due to their high sensitivity, specificity, simplicity of operation, and low cost. The sex pheromone of *Dendrolimus* spp. has been used in monitoring since the 1970s. Population monitoring in low-density forest areas can directly ascertain the size of an insect population in the field (Wen et al., 2000), but different formula should be used for high density *Dendrolimus* populations (Du et al., 2016).

## 3 Molecular Mechanisms of Sex Pheromone Recognition in *Dendrolimus* spp.

### 3.1 Sex Pheromone Recognition–Related Genes Identification

The pheromone recognition system of moths is very developed (Vogt and Riddiford, 1981). *Dendrolimus* are moth insect, and study on their pheromone recognition mechanisms can provide theoretical guidance for the development of efficient pest behavior regulation technology. The sex pheromone recognition system of pine moths is well developed, and study of their pheromone recognition mechanisms can provide theoretical guidance for the development of efficient population regulation technology.

At least six gene families involve in the detection of volatile and pheromone in insects, including three receptor families and two binding protein families (Odorant binding protein, OBP, and chemosensory protein, CSP), and the sensory neuron membrane proteins (SNMPs) (Su et al., 2009). The three receptor families are expressed in insect olfactory sensory neurons, including odorant receptors (OR) (Touhara and Vosshall, 2009), ionotropic receptors (IR) (Benton et al., 2009), and gustatory receptors (GR) (Agnihotri et al., 2016). Olfaction-related genes exhibit large sequence diversity, and identification of large repertoires of olfactory genes by purely homology-based methods is inefficient. Transcriptome researches greatly assisted the pheromone recognition related genes identification and analysis of *Dendrolimus* species.

Using transcriptome from different developmental stages and tissues, a complete sequence and expression profile of olfactory recognition related genes in *D. punctatus* was constructed (Zhang et al., 2017). Identification and comparison of olfactory recognition genes of *D. kikuchii* and *D. houi* indicated that odorant binding proteins (OBPs) and olfactory receptors (ORs) are the molecular basis of olfactory recognition differences between species (Zhang et al., 2014b). In order to solve the difficulty in identifying the pheromone receptor of *D. punctatus*, the antennae transcriptome of different mating stages of this species were compared and analyzed, and the candidate genes of sex pheromone receptor were found, which provided a solid foundation for the subsequent functional analysis of sex pheromone and the discovery of the new origin of pheromone receptor genes (Zhang et al., 2018).

### 3.2 Function Researches of Sex Pheromone Recognition Related Genes

Among the large number of chemosensory genes in insects (Vosshall and Stocker, 2007; Zhang et al., 2017; Zhang et al., 2018), there are two types of specific pheromone recognition genes: 1) pheromone binding proteins (PBPs) (Große-Wilde et al., 2006; Guo et al., 2012), which selectively bind and transport pheromone molecules to the dendrites of olfactory sensilla; 2) pheromone receptors (PRs) (Krieger et al., 2004; Leary et al., 2012; Zhang et al., 2015), which transform the chemical signals into specific neural electric signals. Therefore, the functional analysis of PBPs and PRs are the key to elucidating the pheromone molecular recognition mechanism of *Dendrolimus* spp.

#### 3.2.1 PBP Genes Function Researches

**FIGURE 3:**
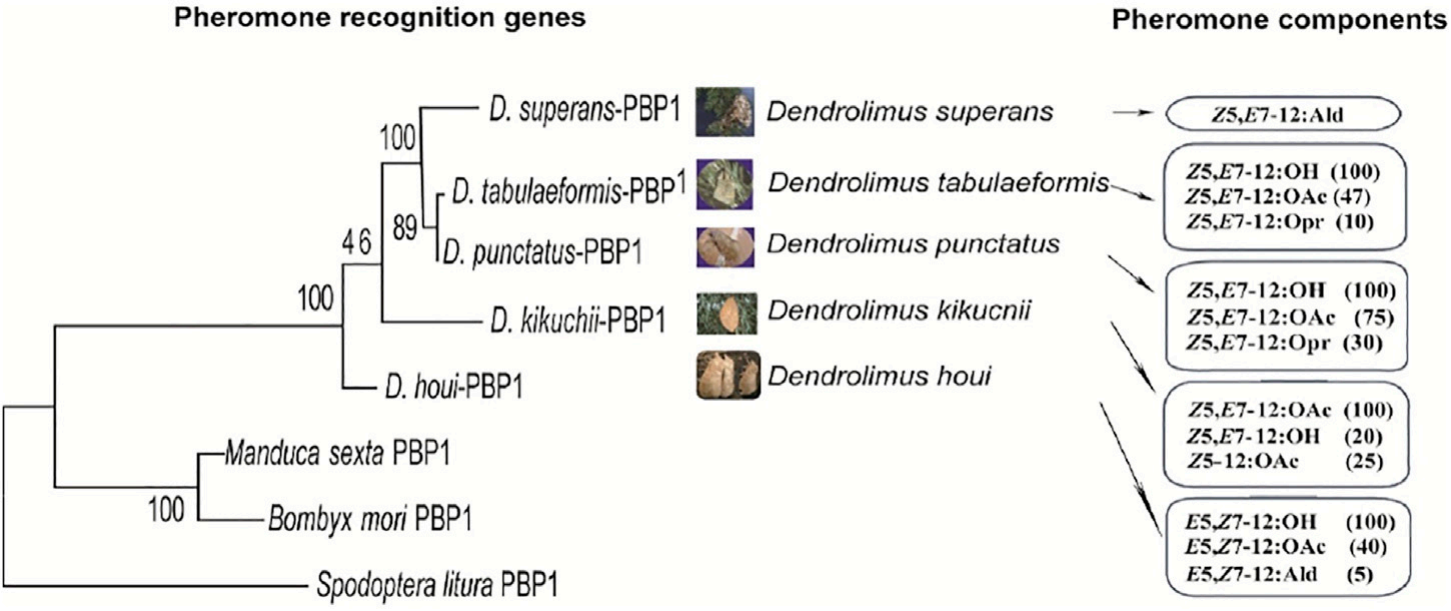
Comparison analysis of phylogenetic relationship of PBP1 and pheromone compound structures evolving in five *Dendrolimus* species. The PBP1 maximum likelihood tree was based on amino acid sequences, bootstrap values from 10,000 replications.

Insect sex pheromones generally consist of 2–3 components at a particular ratio. Research into the function of PBPs obtained from *D. kikuchii* showed that the lower the proportion of a certain pheromone component, the higher the binding efficiency of PBPs to this component (Zhang et al., 2016). The functions of the PBP of *D. tabulaeformis* demonstrated the characteristics of inverse proportion recognition (Zhang et al., 2014c). Inverse proportion recognition might be an important way to recognize the minor sex pheromone components in *Dendrolimus* species. The chemical structure of sex pheromones changes gradually in several important *Dendrolimus* species, as described in Section 2. Phylogenetic analysis shows that the evolution of the PBP genes sequences associates well with the structural changes of pheromone components (Figure 3), indicating that the olfactory recognition genes and sex pheromone structures of *Dendrolimus* species are coevolutionary (Zhang S.-F. et al., 2014).

#### 3.2.2 PR Genes Function Researches

In comparison with general odorant receptors (ORs), the sequences of PRs in moths are relatively conserved and tend to be clustered into the same branch (also called the PR branch) on the evolutionary tree. However, we found that none of the ORs of *Dendrolimus* clustered into the traditional PRs branch, indicating the PRs of *Dendrolimus* spp. have unique sequence characteristics. We cannot find PR candidates of *Dendrolimus* spp. by phylogenetic analysis of ORs. To solve this problem, a new method to identify the PRs gene of *Dendrolimus* spp. was developed, by combining the mating behavior process with gene expression patterns (Zhang et al., 2018). The ORs correspond with identified mating behaviors, and specifically expressed in the antennae of male *D. punctatus*, were selected as PR candidates. Next, six candidate PRs and Orco were cloned from *D. punctatus* and each of the six genes was expressed with an Orco gene found in *Xenopus* oocytes—this gene was physiologically tested with pheromone compounds and analogs of *Dendrolimus* spp. using two-electrode voltage-clamp recording methods. Finally, PR45 and PR46 were successfully identified as two sex pheromone receptors.

**FIGURE 4:**
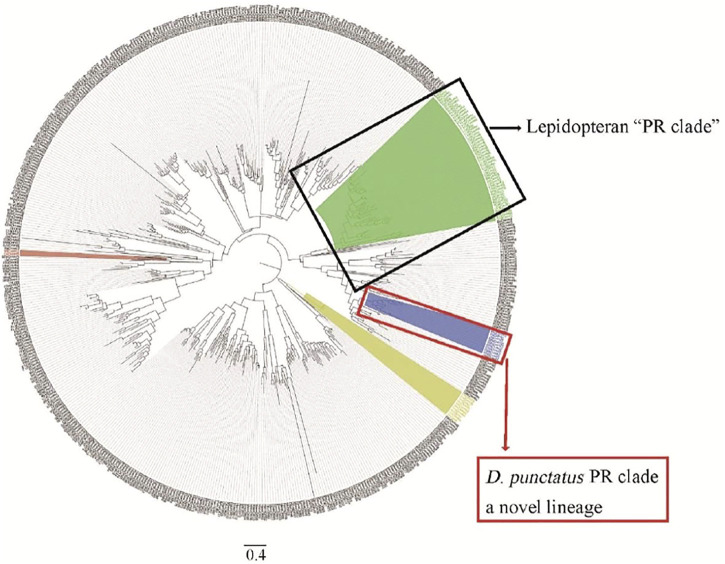
Phylogenetic analysis of ORs from *Dendrolimus* spp. and other lepidopteran insects, which indicates a new lineage of PRs. Dpun: *D. punctatus*, Bmor: *Bombyx mori*, Hvir: *Heliothis virescens*, Harm: *Helicoverpa armigera*, Hass: *Helicoverpa assulta*, Msex: *Manduca sexta*, Slit: *Spodoptera litura*, Ofur: *Ostrinia furnacalis*, Esem: *Eriocrania semipurpurella*, and Epos: *Epiphyas postvittana*. The clade of traditional PR clade is highlighted in green, conserved Orco in yellow, and *D. punctatus* in blue. Part of the picture comes from Shen et al. (2020).

Phylogenetic tree analysis showed that *D. punctatus* PRs were located in a more distinct clade than other lepidopteran PRs, and motif analysis of PRs showed differences between *Dendrolimus* spp. and other tested moth species (Shen et al., 2020). Therefore, our work found a novel lineage of PRs tuned to Type I pheromones in Lepidoptera (Shen et al., 2020) (Figure 4). Genome and mitochondrial phylogenies both indicate that *Dendrolimus* spp. (Lasiocampidae) is close to *Bombyx mori* (Bombycoidea) (Qin et al., 2019; Zhang et al., 2020), but *D. punctatus* PRs appear to diverge more than expected. Maybe the reproductive systems of insects are under larger selection pressure, and these driven accelerating divergence of PR genes in Lepidoptera. Evolution of moths reproductive system is still a mystery, and more work on genetic divergence of close related species are need in the future (Groot et al., 2016).

As *Dendrolimus* species use same or very similar chemicals as sex pheromones, it is unclear that how PRs recognize and discriminate pheromones in closely related *Dendrolimus* species, and this deserves to be further studied.

## 4 Conclusion and Prospects

Remarkable progress has been made in the identification and recognition of the sex pheromone of *Dendrolimus* spp., which provides a basis for further theoretical and practical research. First, the pheromone components of several main species of *Dendrolimus* have been identified, and the sex pheromone components of different *Dendrolimus* species are gradually changed. Second, inverse proportion recognition may be an important way for PBPs to recognize the trace sex pheromone component of *Dendrolimus* spp. Third, the evolution of the sequences of their PBP genes is in good agreement with the trends in structural changes of the sex pheromone components of the major *Dendrolimus* species, which reveals that the olfactory recognition genes and pheromone structures of *Dendrolimus* evolved synergistically. Fourth, PRs of *Dendrolimus* spp. occupy a novel lineage of PRs tuned to Type I pheromones in Lepidoptera. However, more work on pheromone recognition and discrimination of *Dendrolimus* are needed. For example, how closely related *Dendrolimus* species recognize their own sex pheromones accurately (not similar pheromones from closely related speces) and direct mating with homospecies? Does PBPs and PRs sequence mutations or different subsequent neural single integration process responsible for pheromone discrimination among closely related species? This is not only important for *Dendrolimus* but is likely to reveal some of the secrets still hidden in many other sister insect species.
